# Clinical presentation and persistent symptoms in patients at a post-COVID-19 clinic in Ghana

**DOI:** 10.11604/pamj.2023.45.44.38778

**Published:** 2023-05-17

**Authors:** Hanson Gabriel Nuamah, Ebenezer Oduro-Mensah, Joseph Oliver-Commey

**Affiliations:** 1 Department of Epidemiology and Disease Control, School of Public Health, University of Ghana, Accra, Ghana,; 2 National COVID-19 Treatment Centre, Ga East Municipal Hospital, Ghana Health Service, Kwabenya, Ghana,; 3 Ghana Infectious Disease Center, Ga East Municipal Hospital, Kwabenya, Ghana

**Keywords:** COVID-19, chest pain, clinical presentation, symptoms, Ghana

## Abstract

**Introduction:**

on March 11^th^, 2020, the World Health Organization recognized COVID-19 as a pandemic. By March 31^st^, 2021, the Ghana Health Service had recorded a cumulative 90,782 positive cases and 748 deaths in the country. Despite the significant resources and efforts being put into containing and treating individuals with COVID-19, there is a lack of information within sub-Saharan Africa on clinical presentations and factors associated with experiencing persistent symptoms of COVID-19.

**Methods:**

in this retrospective study, we collected data obtained from patients with COVID-19 who were discharged from the post-COVID-19 clinic at the Ga East Municipal Hospital, Ghana, between April 1^st^, 2020, and March 31^st^, 2021, to assess clinical presentations and identify predictors of COVID-19 symptoms that persist beyond 14 days from the onset of the symptom.

**Results:**

of the 253 patients who experienced symptoms of COVID-19, 81 (32.0%) experienced symptoms that persisted beyond 14 days. Cough (64.0%), headache (38.7%), and chest pain (28.1%) were the most common symptoms. After adjusting for covariates, the odds of patients presenting with COVID-19 symptoms that persist beyond 14 days are 98% higher among patients who experienced chest pain compared to those who did not and 2% increased for each additional year of their age.

**Conclusion:**

patient´s age and experiencing chest pain were significant predictors of symptoms that persist beyond 14 days. The findings of our study highlight the need to continue to monitor and care for individuals with identified predictors of experiencing persistent symptoms of COVID-19.

## Introduction

In December 2019, a series of unusual pneumonia cases were reported in Wuhan, China. The cause of the outbreak was determined to be a novel coronavirus, referred to as COVID-19 [1]. On March 11^th^, 2020, the World Health Organization (WHO) declared COVID-19 a pandemic with significantly higher infection and mortality rates compared to its predecessors, including severe acute respiratory syndrome (SARS) and Middle East respiratory syndrome (MERS) [2]. At the end of March 2021, the WHO had recorded a cumulative of 128,197,827 confirmed cases, 3,092,693 of which were recorded in Africa [3]. By March 31^st^, 2021, the Ghana Health Service had recorded a cumulative 90,782 positive cases and 748 deaths in the country [4].

COVID-19 has an unprecedented effect on the immune system and triggers a sudden upregulation of different pro-inflammatory cytokines [5]. The clinical characteristics of COVID-19 are well established. However, studies conducted on different continents reported variations in the most common symptoms [6-8]. Although most patients with COVID-19 recover within weeks of illness, studies have highlighted cases in which patients report symptoms of COVID-19 persisting for several weeks after being infected with the virus [9,10]. Symptoms that include shortness of breath, fatigue, and sleep disorders are symptoms of COVID-19 reported to persist in patients who have recovered from the disease [9,10]. Other similar follow-up studies have identified anosmia and diarrhea [11], age, and sex [12], as well as the severity of COVID-19 [13] as risk factors associated with persistent symptoms of COVID-19. The findings of these studies suggest that there may be additional demographic and clinical factors that may predict individuals who are at risk of experiencing persistent symptoms.

As new cases and recoveries from COVID-19 continue to emerge, identifying the proportion of recovered individuals suffering from and those who are at greater risk of experiencing persistent symptoms will become critical for the development of effective treatment and management strategies. In this study, we evaluated the clinical presentation and persistent symptoms in patients who attended a post-COVID-19 clinic in Ghana. Specifically, we carried out this study to identify the proportion of individuals experiencing persistent symptoms of COVID-19 and assess the associations between the demographic characteristics of the patient, the clinical characteristics, and the persistent symptoms of COVID-19.

## Methods

**Study design:** this is a one-year retrospective cohort study.

**Setting:** this study involved analyzing archived data of COVID-19 patients who were discharged from the post-COVID-19 clinic at the Ga East Municipal Hospital, Ghana, between April 2020 and March 2021. This facility was designated as the national COVID-19 treatment center in Ghana, receiving COVID-19 positive patients from both the Kotoka International Airport and the local community from March 2020 until April 2021. The 100-bed facility is also equipped with a 4-bed high dependency unit/intensive care unit and has a unit for post-COVID-19 review.

**Participants:** our study population consisted of all patients admitted to the facility who were confirmed by polymerase chain reaction (PCR) for COVID-19. Patients were followed up daily until they were declared fully recovered by the medical staffs and discharged from the facility.

**Variables:** we used the term “persistent symptoms” to describe patients with self-reported COVID-19-like symptoms that persisted beyond 14 days after symptom onset. Furthermore, we used the term “experienced” for all self-reported symptoms from the onset of the symptom until the last report from the patient. The presence or absence of persistent symptoms was the dependent variable. Independent variables were demographic and clinical characteristics, including patient age, sex, nationality, underlying health conditions, as well as clinical information including admission period, worst COVID-19 severity recorded during admission, and symptoms.

**Data sources:** self-reported age, sex, nationality, underlying health conditions, and symptoms were collected from the hospital records. Clinical information on the patient´s admission period, worst COVID-19 severity during admission documented by the medical staffs were also collected from the medical records. Disease severity was categorized as mild, moderate, or severe based on the standard WHO classification of COVID-19 severity.

**Bias:** potential recall bias was kept to a minimum since patients, while on admission, reported their symptoms to the doctors daily.

**Study size:** to focus our assessment on patients brought in from the local community, we excluded COVID-19 positive travelers who were brought in from Kotoka International Airport. We also excluded patients who were asymptomatic from diagnosis until they were declared fully recovered, and those discharged from the facility to continue home-based treatment.

**Quantitative variables:** in our study, patient age was grouped as those ≤19, 20-39, 40-64, and ≥65 years. These categories were used to present age distribution and compare differences in the patient´s characteristics with the presence or absence of persistent symptoms. Self-reported age (continuous variable) was used in the logistic regression analysis.

**Statistical methods:** we collected the archived medical records of patients using a structured data collection form and exported the data to Stata 16.1 (StataCorp, College Station, Texas, USA) for analysis. We had no missing data from those included in the study. The continuous variables are presented as medians and interquartile range (IQR), while categorical variables are presented as frequency and percentages. We used Chi-square test or Fisher´s exact test to analyze differences between dependent and independent variables as well as a logistic regression to assess factors associated with developing symptoms of COVID-19 that persisted beyond 14 days. The results of the logistic regression analysis are reported as crude odds ratio (COR) and adjusted odds ratio (AOR). Backward elimination was used for sequential removal of non-significant variables (p ≥ 0.05) from the adjusted model. We set the confidence interval (CI) at 95% and two-tailed p-values <0.05 were considered significant.

**Ethical approval:** the research protocol was approved by the Ghana Health Service Ethics Review Committee (Protocol identification number: GHS-ERC: 012/07/21). All information gathered was treated with strict confidentiality.

## Results

**Participants:** from April 1^st^, 2020, to March 31^st^, 2021, 350 COVID-19 positive patients with no travel history for two weeks were admitted to the post-COVID-19 clinic at the Ga East Municipal Hospital and stayed until discharged after being declared fully recovered. Of the 350 patients, 253 (72.3%) reported one or more symptoms of COVID-19 during admission. We excluded the remaining 97 (27.7%) asymptomatic patients from the study.

**Descriptive data:** the characteristics of the 253 participants are summarized in Table 1. Their median age was 45 years (IQR = 28 - 57 years), with a large percentage (55.0%) of them over 40 years. A slight majority (59.3%) of the patients were male, while most (94.9%) of all patients were Ghanaian nationals. Almost half (46.6%) of the patients reported having no known comorbidities. During the study period, 46.6% of patients were admitted before the reopening of Kotoka International Airport in September 2020, while the other 53.4% were admitted after the reopening of the airport.

**Table 1:** demographic characteristics of patients with symptoms

Characteristics	Frequency	
	**n**	**(%)**
**Age**		
≤19 years	37	14.6
20-39 years	77	30.4
40-64 years	107	42.3
≥65 years	32	12.7
**Sex**		
Male	150	59.3
Female	103	40.7
**Nationality**		
Ghanaians	240	94.9
Non-Ghanaians	13	5.1
**Comorbidities**		
Diabetes	53	21.0
Hypertension	93	36.8
Other	49	19.4
No known comorbidities	118	46.6
**Admission period**		
Before airport reopening	118	46.6
After airport reopening	135	53.4
**Severity**		
Mild	154	60.9
Moderate	60	23.7
Severe	39	15.4

n: number of patients

The median time between symptom onset and the last reported symptom was 10 days (IQR = 6 - 17 days). The most common symptoms reported by the patients were cough (64.0%), headache (38.7%), and chest pain (28.1%). Cough (45.5%), headache (24.5%), and shortness of breath (17.4%) were the first symptoms reported, while cough (41.1%), headache (11.9%), and chest pain (9.9%) were the last (Table 2).

**Table 2:** clinical presentation of patients by symptoms

Clinical presentation	Symptoms					
	**Reported**		**First reported**		**Last reported**	
	**n**	**(%)**	**n**	**(%)**	**n**	**(%)**
Cough	162	(64.0)	115	(45.5)	104	(41.1)
Headache	98	(38.7)	62	(24.5)	30	(11.9)
Chest pain	71	(28.1)	35	(13.8)	25	(9.9)
Shortness of breath	70	(27.7)	44	(17.4)	24	(9.5)
Sore throat	53	(21.0)	33	(13.0)	24	(9.5)
Fever (+37.2°C)	52	(20.6)	25	(9.9)	12	(4.7)
Loss of smell or taste	50	(19.8)	33	(13.0)	23	(9.1)

n: number of patients

**Outcome data:** we recorded 81 (32.0%) patients with COVID-19 symptoms that persisted beyond 14 days from symptom onset. A significant percentage (40.6%) of patients over 64 years of age reported that their symptoms persist beyond 14 days compared to patients under 20 years of age (5.4%); p<0.01, Chi-square test. A large percentage (39.8%) of hypertensive patients reported persistent symptoms beyond 14 days compared to patients without known hypertension (27.5%); p = 0.04, Chi-square test. A significant percentage (42.3%) of patients who experienced chest pain reported that their symptoms persisted beyond 14 days compared to those who did not (28.0%); p = 0.03, Chi-square test (Table 3).

**Table 3:** relationship between patient characteristics and persistent symptoms

Characteristics	Symptoms				P-value
	**Resolved within 14 days**		**Persisted beyond 14 days**		
	**n**	**(%)**	**n**	**(%)**	
**Age**					**< 0.01**
≤19yrs	35	(94.6)	2	(5.4)	
20-39yrs	51	(66.2)	26	(33.8)	
40-64yrs	67	(62.6)	40	(37.4)	
≥65yrs	19	(59.4)	13	(40.6)	
**Sex**					**0.59**
Male	100	(66.7)	50	(33.3)	
Female	72	(69.9)	31	(30.1)	
**Nationality**					**0.24**
Ghanaians	161	(67.1)	79	(32.9)	
Non-Ghanaians	11	(84.6)	2	(15.4)	
Comorbidities					
**Diabetes**					**0.50**
Absent	138	(69.0)	62	(31.0)	
Present	34	(64.2)	19	(35.9)	
**Hypertension**					**0.04**
Absent	116	(72.5)	44	(27.5)	
Present	56	(60.2)	37	(39.8)	
**Other comorbidities**					**0.82**
Absent	138	(67.6)	66	(32.4)	
Present	34	(69.4)	15	(30.6)	
**Admission period**					**0.05**
Before airport reopening	73	(61.9)	45	(38.1)	
After airport reopening	99	(73.3)	36	(26.7)	
**Severity**					**0.14**
Mild	107	(69.5)	47	(30.5)	
Moderate	35	(58.3)	25	(41.7)	
Severe	30	(76.9)	9	(23.1)	
**Symptoms**					
**Cough**					**0.15**
Absent	67	(73.6)	24	(26.4)	
Present	105	(64.8)	57	(35.2)	
**Headache**					**0.86**
Absent	106	(68.4)	49	(31.6)	
Present	66	(67.3)	32	(32.7)	
**Chest pain**					**0.03**
Absent	131	(72.0)	51	(28.0)	
Present	41	(57.7)	30	(42.3)	
**Shortness of breath**					**0.63**
Absent	126	(68.8)	57	(31.2)	
Present	46	(65.7)	24	(34.3)	
**Fever (+37.2°C)**					**0.65**
Absent	138	(68.7)	63	(31.3)	
Present	34	(65.4)	18	(34.6)	
**Sore throat**					**0.99**
Absent	136	(68.0)	64	(32.0)	
Present	36	(67.9)	17	(32.1)	
**Loss of smell or taste**					**0.87**
Absent	137	(67.5)	66	(32.5)	
Present	35	(70.0)	15	(30.0)	

n: number of patients; differences assessed with Chi-square test or Fisher’s exact test

**Main results:** after adjusting for variables with p-value <0.05 from the crude odds ratio analysis, the results revealed that the patient´s age and experiencing chest pain were significant predictors of symptoms that persist beyond 14 days (Table 4). The odds of patients experiencing COVID-19 symptoms that persist beyond 14 days are 98% higher among patients who reported chest pain compared to those who did not (adjusted odds ratio (AOR) = 1.98, 95% CI 1.10-3.55, p = 0.02). Furthermore, the odds of experiencing COVID-19 symptoms that persist beyond 14 days are increased by 2% for each additional year of their age (AOR = 1.02, 95% CI 1.00-1.04, p = 0.02).

**Table 4:** characteristics associated with symptoms persisting beyond 14 days

Characteristics	Symptoms persisting beyond 14 days			
	**COR (95% CI)**	**P-Value**	**AOR (95% CI)**	**P-Value**
Age	1.02 (1.01-1.04)	<0.01	1.02 (1.00-1.04)	0.02
**Sex**				
Male	Reference			
Female	0.86 (0.50-1.48)	0.59	-	
**Nationality**				
Ghanaians	Reference			
Non-Ghanaians	0.37 (0.08-1.71)	0.20	-	
**Comorbidities**				
No known comorbidities	Reference			
Diabetes	1.24 (0.66-2.35)	0.50	-	
Hypertension	1.74 (1.01-2.99)	0.04	1.20 (0.62-2.33)	0.58
Other comorbidities	0.92 (0.47-1.81)	0.82	-	
**Admission period**				
Before airport reopening	Reference			
After airport reopening	0.59 (0.35-1.00)	0.05	-	
**Severity**				
Mild	Reference			
Moderate	1.75 (0.96-3.18)	0.07	-	
Severe	0.59 (0.27-1.31)	0.20	-	
**Symptoms**				
Absent	Reference			
Cough	1.52 (0.86-2.67)	0.15	-	
Headache	1.05 (0.61-1.80)	0.86	-	
Chest pain	1.88 (1.06-3.33)	0.03	1.98 (1.10-3.55)	0.02
Shortness of breath	1.15 (0.64-2.07)	0.63	-	
Fever (+37.2°C)	1.16 (0.61-2.21)	0.65	-	
Sore throat	1.00 (0.52-1.92)	0.99	-	
Loss of smell or taste	0.89 (0.45-1.74)	0.73	-	

CI: confidence interval; COR: crude odds ratio; AOR: adjusted odds ratio; variables included in the adjusted model: age, hypertension, and chest pain

## Discussion

Our study assessed the clinical presentation of COVID-19 and factors associated with the experience of persistent symptoms. Our findings on the most common symptoms of COVID-19 were consistent with the results of an earlier published study in Accra, Ghana, which also identified cough in over half (57.5%) of their symptomatic patients [14]. However, the low reporting (20.6%) of fever among patients in this study suggests that noncontact infrared thermometers are not suitable as a stand-alone screening tool for COVID-19 positive patients.

We identified age as a significant predictor of symptoms that persist beyond 14 days. This finding is consistent with a previous study that analyzed 4,182 cases of COVID-19 and reported that age is significantly associated with symptoms that persist beyond 28 days [12]. Experiencing chest pain was also identified as a significant predictor of persistent disease symptoms. The chest pain reported in recovered patients may be attributed to inflammation of the pleura [1].

One limitation of our study was the dependence on self-reported symptoms. However, the potential recall bias was kept to a minimum since patients, while on admission, reported their symptoms to the doctors daily. Another limitation was the inability to distinguish between possible variants of COVID-19 within the study population, which may have influenced the study findings.

In contrast to a previous retrospective study of 274 COVID-19 survivors from the Lagos State COVID-19 Outpatient Clinic, we did not identify the severity of COVID-19 as a predictor of experiencing persistent symptoms [13]. The findings of our study highlight the need to continue to monitor and care for individuals with identified predictors of experiencing persistent symptoms of COVID-19.

## Conclusion

Cough (64.0%), headache (38.7%), and chest pain (28.1%) were the most common symptoms. More than a quarter (32.0%) of the patients experienced symptoms of COVID-19 that persisted beyond 14 days after the onset of the symptoms. After adjusting for covariates, the odds of patients presenting with COVID-19 symptoms that persist beyond 14 days are 98% higher among patients who experienced chest pain compared to those who did not and 2% increased for each additional year of their age.

### What is known about this topic

- *Shortness of breath, fatigue, sleep disorders, anosmia, and diarrhea are symptoms of COVID-19 reported to persist in some patients who have recovered from the disease;*
- *Age and sex are risk factors associated with persistent COVID-19 symptoms*.

### What this study adds

- *A large percentage (32.0%) of patients presented with COVID-19 symptoms that persisted beyond 14 days from symptom onset;*
- *Cough (64.0%), headache (38.7%), and chest pain (28.1%) were the most common symptoms in this cohort;*
- *The odds of patients presenting with COVID-19 symptoms that persist beyond 14 days are 98% higher among patients who experienced chest pain compared to those who did not*.
